# Soil-Mulching Treatment Enhances the Content of Stilbene in Grape Berries: A Transcriptomic and Metabolomic Analysis

**DOI:** 10.3390/foods13193208

**Published:** 2024-10-09

**Authors:** Bo Wang, Weimin Wu, Zhuangwei Wang, Zhenxiao Chen, Xicheng Wang

**Affiliations:** Institute of Pomology, Jiangsu Academy of Agricultural Sciences/Jiangsu Key Laboratory for Horticultural Crop Genetic Improvement, No. 50 Zhongling Street, Nanjing 210014, China; wb@jaas.ac.cn (B.W.);

**Keywords:** mulching, grape, stilbene, transcriptome, metabolomics, berry quality, weighted gene co-expression network analysis (WGCNA)

## Abstract

Soil mulching is a useful agronomic practice that promotes early fruit maturation and affects fruit quality. However, the regulatory mechanism of fruit metabolites under soil-mulching treatments remains unknown. In this study, variations in the gene sets and metabolites of grape berries after mulching (rice straw + felt + plastic film) using transcriptome and metagenomic sequencing were investigated. The results of the cluster analysis and orthogonal projection to latent structures discriminant analysis of the metabolites showed a difference between the mulching and control groups, as did the principal component analysis results for the transcriptome. In total, 36 differentially expressed metabolites were identified, of which 10 (resveratrol, ampelopsin F, piceid, 3,4′-dihydroxy-5-methoxystilbene, ε-viniferin, trans resveratrol, epsilon-viniferin, 3′-hydroxypterostilbene, 1-methyl-resveratrol, and pterostil-bene) were stilbenes. Their content increased after mulching, indicating that stilbene synthase activity increased after mulching. The weighted gene co-expression network analysis revealed that the turquoise and blue modules were positively and negatively related to stilbene compounds. The network analysis identified two seed genes (VIT_09s0054g00610, VIT_13s0156g00260) and two transcription factors (VIT_13s0156g00260, VIT_02s0025g04590). Overall, soil mulching promoted the accumulation of stilbene compounds in grapes, and the results provided key genetic information for further studies.

## 1. Introduction

Grapevines are among the most economically important fruit trees worldwide [1]. In most southern areas of China, the maturity period of table grapes is from July to August, whereas in most northern areas of China, the maturity period is in September. The concentration of maturity often decreases the price of table grapes. Early maturation is a key agronomic trait of plants; however, the common breeding procedure for early maturing varieties is challenging in fruit tree breeding [2,3]. Promoting early maturity through agronomic practices such as dormancy breaking or soil mulching—which can help raise the selling price of grapes, reduce the use of pesticides, and avoid the hazard of typhoons—is common and effective for boosting production [4,5].

Yield and quality are important factors for producers when promoting early mature cultivation systems. Previous studies have shown that crop or fruit yield, nutritional content, and flavor change in an early-maturity cultivation model [6,7]. For example, a combination of chemical application treatments significantly advanced budbreak, and apple fruit quality and yield increased after treatment [8]. Another study showed that different plastic-film mulch treatments accelerated tomato ripening and changed yield and quality [9,10]. Although agronomic practices for promoting early maturity can affect plant growth and quality performance, the underlying regulatory mechanisms remain unclear.

Stilbenes are natural phenolic compounds synthesized in plants that mainly act as phytoalexins in response to abiotic- or biotic-stress conditions [11,12]. In addition, previous studies have demonstrated that stilbenes possess various health-promoting properties, including antioxidant, anti-aging, and anticancer effects [13]. Stilbene occurred in limited plant species because stilbene synthase (STS), the key enzyme in stilbene biosynthesis, is not commonly distributed in all plant species [14]. Table grapes or wine are primary sources of stilbenes for humans [15]. The content of stilbenes in grapes can be affected by factors such as grape variety, growth stage, agronomy practices, and environmental conditions [16,17]. It remains unclear how the content of stilbenes in grapes varies under early maturing cultivation systems.

A weighted gene co-expression network analysis (WGCNA) was used to determine modules of highly correlated genes and identify gene modules related to external phenotypic traits [18]. This is useful for identifying genes associated with numerous gene sets and target traits. Thus far, it has been used to screen for key genes associated with plant development and quality as well as abiotic and biotic stresses [19,20,21,22]. Sheng et al. [23] used transcriptome sequencing to identify gene co-expression patterns of gene networks highly related to sesame plant height; a module showed a significantly positive relationship with plant height, and a hub gene in the regulation of plant height was found using WGCNA. Wang et al. [19] used metabolome- and genome-wide transcriptome analyses to reveal flavor formation in kiwifruit, and WGCNA was conducted to investigate the co-expression patterns of differentially expressed genes (DEGs), finding that gene sets in three modules were associated with flavor-associated metabolites. The same analysis method has also been used to investigate key gene modules associated with heat stress, drought stress, and powdery-mildew resistance [19,24,25].

Our previous study found that grape ripening occurred 5 to 7 days earlier in the soil mulch group than in the control group, but changes in fruit quality were not evaluated. The present study determined fruit quality during the maturation period using conventional physical testing and transcriptomic and metabolomic methods. This study aimed to reveal and explain the changes in grape quality during soil mulching.

## 2. Materials and Methods

### 2.1. Plant Materials and Field Experiment

The field experiment was conducted in a vineyard within a plastic greenhouse. The study site was located at the Jiangsu Academy of Agricultural Sciences, Nanjing, Jiangsu Province, China (32°02′ N, 118°52′ E, approximately above sea level, with an average annual precipitation of 1106 mm, frost-free 225 d). Zijinhongxia (*Vitis vinifera* L.) was used as the plant material in this study, with a planting density of 1.8 m between lines. There were two treatments with three replicates in the present study: (1) conventional tillage with no mulching (control) and (2) mulching with rice straw + felt + plastic film (treatment). Each replicate consisted of 3 grapevine trees. The field experiments were conducted between December 2021 and July 2022. In December 2021, the grapevines were spur-pruned. In February 2022, the plastic film was closed, and the grapevines were irrigated. Two days after irrigation, the soil was covered with rice straw (5 cm thickness), and then, an arched shed (0.57 m height, 0.8 m width) was constructed with iron wire (3.5 mm thickness), glass fiber bar (5 mm thickness, 1.5 m length), felt (200 g∙m^2^), and plastic film (0.08 mm).

### 2.2. Plant Sampling

The grape berries were sampled 90 d after flowering. Three biological replicates were obtained, each consisting of 54 berries from 6 clusters. The berries were immediately frozen in liquid nitrogen and stored at −80 °C for ribonucleic acid (RNA) extraction and metagenomic analyses. In addition, fruit quality, including single-berry weight, longitudinal and transverse diameters, total soluble solids, total soluble sugar, and total titratable acid, was determined according to a handbook of plant physiology experiments and specifications for grape germplasm description [26,27]. Stilbene synthase activity was determined using an astragalus synthase (STS) ELISA kit (KIRbio, Beijing, China).

### 2.3. RNA Extraction, High-Throughput Sequencing, and Data Processing and Analysis

Using the RNAprep Pure Plant Kit (TIANGEN, Beijing, China), total RNA was extracted. Its quantity was measured by Nanodrop ND-2000 (Nanodrop technologies, Wilmington, DE, USA). The NEBNext Ultra II FS DNA Library Kit for Illumina^®^ (NEB, Ipswich, MA, USA) was used for complementary deoxyribonucleic acid library construction and then sequencing on Illumina sequencing platform (Metware Biotechnology Co., Ltd., Wuhan, China) [28]. Gene expression was estimated using the fragments per kilobase of transcript per million mapped reads, which was computed using featureCounts v1.6.2/StringTie v1.3.4d [29,30]. The differential expression between the two groups was analyzed using DESeq2 v1.22.1/edgeR v3.24.3 [31,32], and the Benjamini–Hochberg technique was employed to adjust the *p*-values. For varying expression levels, the corrected *p*-value and |log2foldchange| were employed as thresholds. A hypergeometric test was used to perform an enrichment analysis. Pathway units were used in a hypergeometric distribution test for a Kyoto Encyclopedia of Genes and Genomes (KEGG) analysis. Finally, an analysis was conducted using gene ontology (GO).

### 2.4. Metabolomic Analysis

Using a vacuum freeze-dryer (Scientz-100F, Ningbo Scientz Biotechnology Co., Ltd., Ningbo, China), the fruit samples were freeze-dried and then dissolved in 70% methanol solution. Using a UPLC-ESI-MS/MS system (ExionLCTM AD; MS, Applied Biosystems 6500 Q TRAP (ABsciex, Framingham, MA, USA)), the supernatant was analyzed under the analytical conditions specified by [33]. The procedure outlined by [34] was used to identify the differentially expressed metabolites (DEMs). The differences between the control and treatment groups were investigated using an orthogonal projection to latent structures discriminant analysis (OPLS-DA) model.

### 2.5. WGCNA and Correlation Analysis

The software WGCNAv1.69 R was used for the gene co-expression network analysis [35]. The networks were visualized by Cytoscape (3.8.2). Correlations between different modules derived from WGCNA and stilbene compounds were evaluated using Pearson correlation coefficients. The functions of the DEMs were determined using the KEGG database. The functions of the DEGs were annotated using GO and KEGG databases.

## 3. Results

### 3.1. Fruit Appearance and Quality

Mulching treatment did not result in obvious differences in fruit appearance or quality (Table S1). Various parameters including single-berry weight, transverse diameter, soluble solids, soluble sugar, and titratable acid did not show any significant variation after mulching. Only the longitudinal diameter increased under the mulching treatment (Table S1). In both groups, the shape index fell in the range of 1.1–1.3, showing that berry shape did not change under mulching treatment.

### 3.2. Metabolomic Level Analysis of Grape Berries under Mulching Treatment

**Figure 1:**
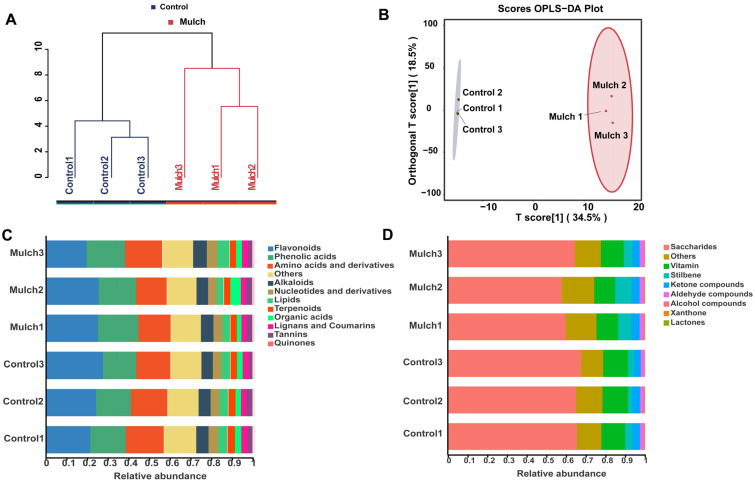
Metabolites of berry fruit in the control and mulch treatment groups: (**A**) Cluster analysis based on Bray–Curtis distances of metabolites; (**B**) OPLS-DA based on Bray–Curtis distances of metabolites; (**C**) Relative abundance of metabolites in Class I and (**D**) others in Class I.

Based on the metagenome results, two distinct distinctions were observed in both cluster analysis and OPLS-DA (Figure 1). The results indicated clear differences between the mulch and control groups. All the metabolites determined were divided into nine classes under Class I, and alkaloid, nucleotide, derivative, and phenolic acid contents were higher in the mulching treatment than in the control (Figure 1C, Table S2). The detected compounds from the other Class I classes were also divided into nine classes, and aldehyde compounds and lactones were more abundant in the mulch treatment than in the control (Figure 1D, Table S3).

**Figure 2:**
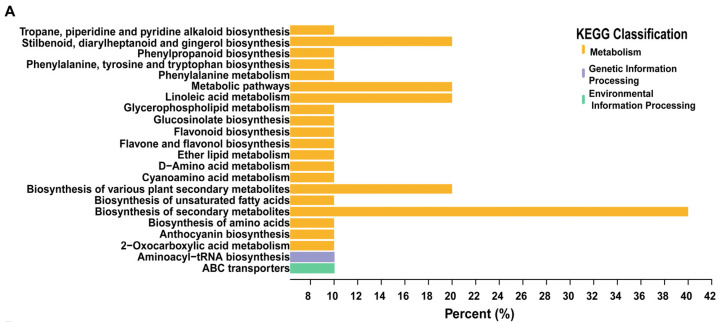

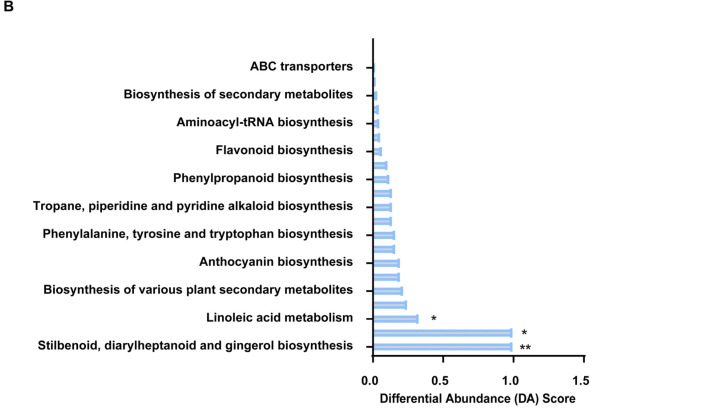
Functions of differentially expressed metabolites (DEMs) in grape berries: (**A**) KEGG annotation pathways of DEMs; (**B**) KEGG pathway analysis of the DEMs. * *p* < 0.05, ** 0.001 < *p* < 0.01.

The metabolites that differed significantly between the control and mulch treatment groups were largely related to metabolism, particularly the biosynthesis of secondary metabolites (Figure 2A). According to the KEGG enrichment results (Figure 2B, Table S4), the differential metabolites were assigned to 23 KEGG pathways, and the top 4 significant pathways were stilbenoid, diarylheptanoid, and gingerol biosynthesis (ko00945); linoleic acid metabolism (ko00591); ether lipid metabolism (ko00565); and the biosynthesis of various plant secondary metabolites (ko00999).

**Figure 3:**
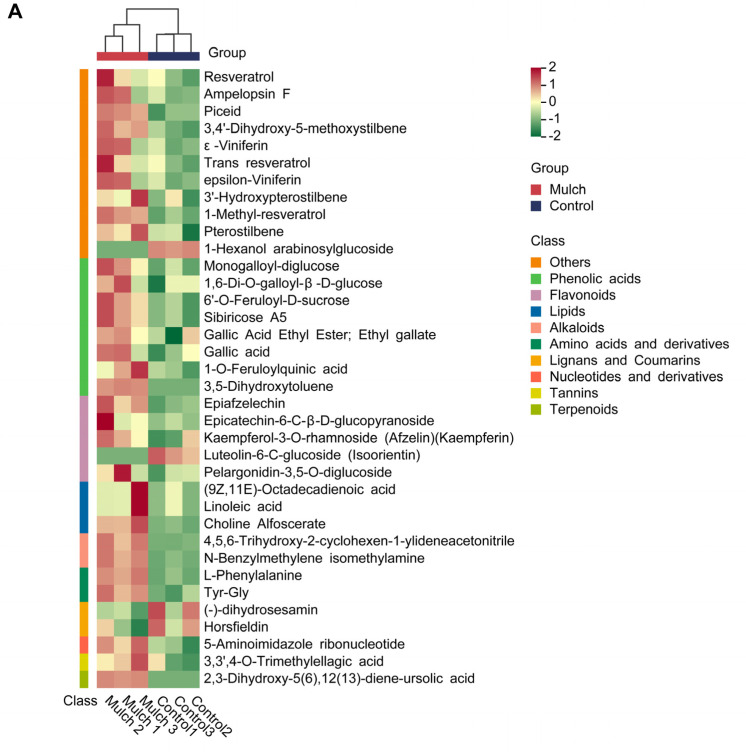

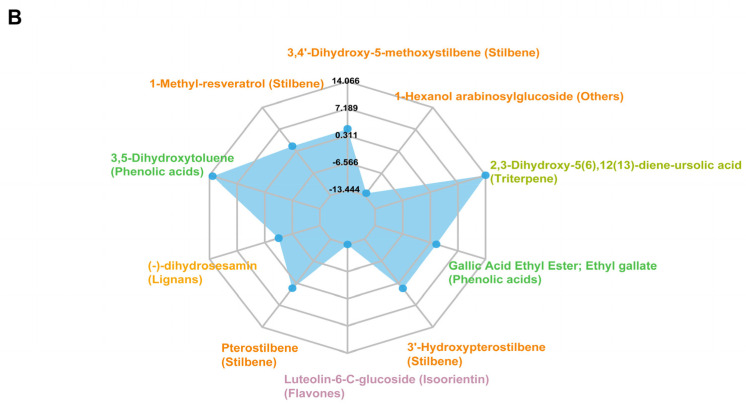
Differentially expressed metabolites (DEMs) in the control and mulch treatment groups: (**A**) Heatmap of the DEMs between the control and mulch treatments. Green indicates downregulated metabolites and red indicates upregulated metabolites; (**B**) Radar charts of the DEMs in the control and mulch treatment groups.

Thirty-six differential metabolites were identified, as shown in the heatmap (Figure 3A). Phenolic acids, flavonoids, and other compounds accounted for 66.67% of the differential metabolites, whereas stilbene metabolites accounted for more than 99% of the other metabolites (Figure 3A). All stilbene metabolites observed (resveratrol, ampelopsin F, piceid, 3,4′-dihydroxy-5-methoxystilbene, ε-viniferin, trans resveratrol, epsilon-viniferin, 3′-hydroxypterostilbene, 1-methyl-resveratrol, and pterostilbene) in this study were higher in the mulching treatment than in the control. Similarly, all phenolic acids (monogalloyl-diglucose, 1,6-Di-O-galloyl-β -D-glucose, 6′-O-feruloyl-D-sucrose, sibiricose A5, gallic acid ethyl ester, ethyl gallate, gallic acid, 1-O-feruloylquinic acid, and 3,5-dihydroxytoluene) were higher in the treatment group than in the control group (Figure 3A). The differential radar chart shows that the five most significant metabolites were 2,3-dihydroxy-5(6),12(13)-diene-ursolic acid, 3,5-dihydroxytoluene, pterostilbene, gallic acid ethyl ester, ethyl gallate, and 3,5-dihydroxytoluene (Figure 3B).

### 3.3. Transcriptomic Level Analysis of Grape Berries between the Control and Mulching Groups

**Figure 4:**
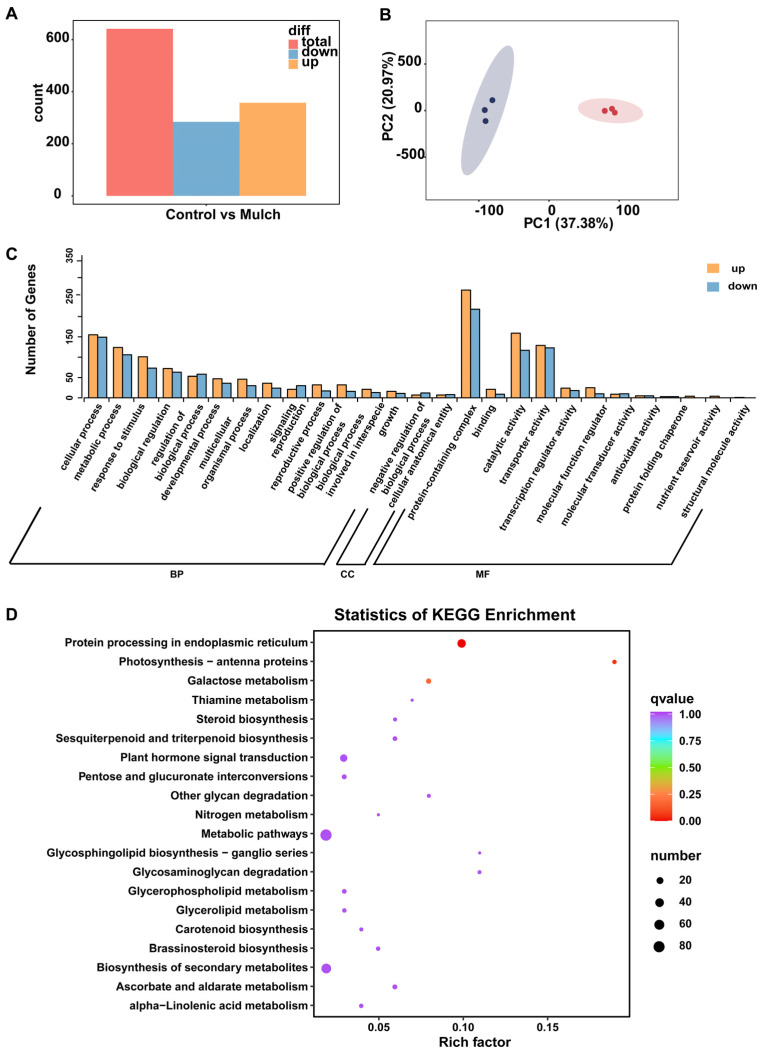
Expressions of berry fruit genes in the control and mulch treatment groups: (**A**) Counts of differently expressed genes in the control and mulch-treated groups; (**B**) PCA of berry fruits based on Bray–Curtis distances; (**C**) Number of upregulated and downregulated genes in the gene ontology (GO) classification; (**D**) KEGG pathways enriched with DEGs.

This study identified 289,253,990 reads from the six treatments; the Q20 rates were all greater than 97%, and the Q30 rates were all greater than 92% (Table S5). As Figure 4A shows, 641 DEGs (upregulated: 357, downregulated: 284) were identified when the control and mulch treatment groups were compared. Based on the PCA results, the control and mulch treatments were grouped into two significantly different clusters (Figure 4B). GO enrichment of the DEGs showed that cellular processes, metabolic processes, responses to stimuli in biological processes (BPs), cellular anatomical entities in cellular components (CCs), and binding and catalytic activities in molecular functions (MFs) were the most likely targets of mulch treatment (Figure 4C). In addition, the KEGG pathway enrichment analysis results (Figure 4D, Table S6) showed that the top five enriched pathways were (ko04141), photosynthesis–antenna proteins (ko00196), galactose metabolism (ko00052), thiamine metabolism (ko00730), and steroid biosynthesis (ko00100). However, only one significantly enriched pathway (protein processing in the endoplasmic reticulum, corrected *p* < 0.001) was identified.

### 3.4. Integrated Transcriptomic and Metabolomic Analysis

#### 3.4.1. KEGG Enrichment Analysis

**Figure 5:**
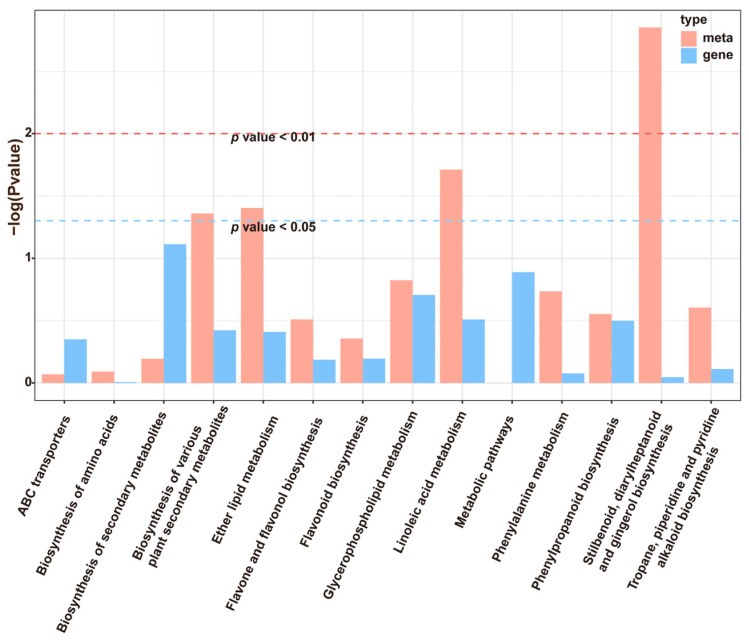
Statistical diagrams of KEGG pathways co-enriched with DEMs and DEGs.

Furthermore, a combination of transcriptome and metabolomic analyses were used to determine the co-enrichment of DEGs and DEMs. The results showed that 14 KEGG pathways were mapped for the enrichment of DEGs and DEMs (Figure 5). Among the DEM-enriched KEGG pathways, four pathways (stilbenoid, diarylheptanoid, gingerol biosynthesis, linoleic acid metabolism, ether lipid metabolism, and biosynthesis of various plant secondary metabolites), stilbenoid, diarylheptanoid, and gingerol biosynthesis were significantly enriched. In addition, the stilbene synthase activity in the mulch treatment was higher than that in the control (Figure 5). These results indicate a greater effect of mulching on metabolites related to stilbene compounds.

#### 3.4.2. Weighted Gene Co-Expression Network Analysis of DEGs and Correlation with Stilbene Compounds

**Figure 6:**
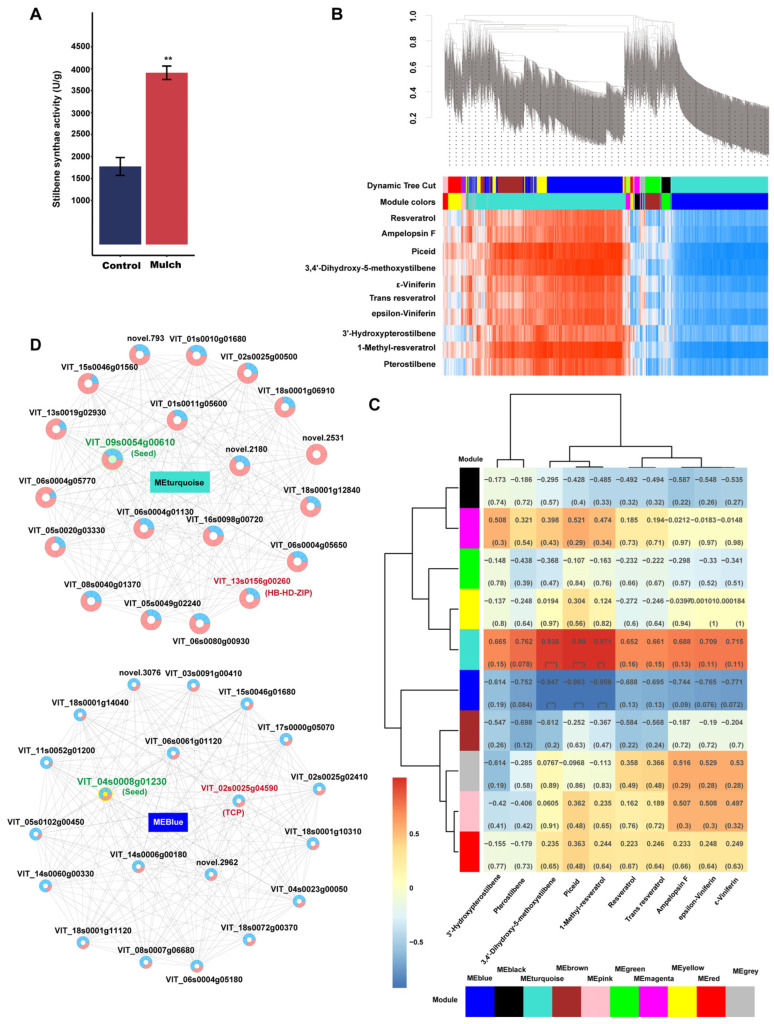
Weighted correlation network analysis modules of all genes established after soil mulch treatment: (**A**) Stilbene activity in the control and mulch treatment groups; (**B**) Tree graphs of 3000 genes by hierarchical clustering of overlapping topological dissimilarities. Red indicates a positive correlation, and blue indicates a negative correlation; (**C**) Heatmap showing module–stilbene compound correlations. Each row corresponds to a module indicated by a different color. Red indicates a positive correlation, and blue indicates a negative correlation; (**D**) Co-expression gene network of MEblue and MEturquoise. Blue indicates gene expression in the control, and pink indicates gene expression in the mulch. * *p* < 0.05, ** 0.001 < *p* < 0.01, *** *p* < 0.001.

To further determine the molecular mechanism of the change in stilbene caused by mulching, 24,760 genes were subjected to WGCNA, which identified 10 co-expression modules (Figure 6A). A module–trait heatmap showed that genes belonging to the turquoise and blue modules were more strongly correlated with stilbene compounds than genes from the other modules (Figure 6A).

Next, a correlation analysis was conducted between the contents of the 10 stilbene compounds and the 10 modules. The correlation analysis results showed that the accumulation of transcripts in turquoise and blue was significantly positively and negatively correlated with stilbene-associated metabolites, including 3,4′-dihydroxy-5-methoxystilbene, piceid, and 1-methyl-resveratrol, respectively (Figure 6B). These results indicate that the genes in these two modules were mainly associated with stilbene compounds. Furthermore, hub gene networks of the turquoise and blue modules were obtained (Figure 6C). The number of hub genes in the turquoise module in the mulching treatment was higher than that in the control, whereas the counts of hub genes in the blue module were higher in the control than in the mulching treatment; VIT_09s0054g00610 was the seed gene in the turquoise module, and VIT_13s0156g00260 was the seed gene in the blue module. In these two modules, transcription factors HB-HD-ZIP TCP (VIT_13s0156g00260) and TCP (VIT_02s0025g04590) were observed.

## 4. Discussion

As a conventional soil management practice, mulching affects not only soil conditions but also plant yield and quality. Previous studies have shown that mulch strategies can enhance fruit production and quality [36]. For example, ref. [37] found that organic and plastic mulches had significant positive effects on pomegranate fruit size, which contributed to an increase in fruit yield, while [38] showed that tomato yield decreased with plastic-film mulching treatment. In the present study, no significant differences were observed in the berry fruit weight. The berry shape index showed that the shapes of the berries were the same in the control and mulch treatment groups. This result was consistent with that reported by [39]. In this study, soluble solids, sugars, and titratable acids were evaluated. Some studies have found that plastic films or a combination of plastic films and straw mulches improve the total soluble-solid content [40,41]. However, another study found that mulched soil in double-layer high tunnels led to lower total soluble solids and total soluble-acid values in tomatoes than those in bare soil [42]. No obvious differences were observed between the three indicators. Therefore, variations in fruit weight, yield, and quality among the different mulching treatments were not always the same, and the treatment period, plant species, and mulching materials might have affected these indicators.

Metagenomic sequencing results showed that the content of several stilbene compounds increased with the mulching treatment (Figure 3). KEGG pathways of the DEMs related to stilbenoid, diarylheptanoid, and gingerol biosynthesis were identified (Figure 2). The results showed that the grapes subjected to the mulching treatment in our study had higher antioxidant properties. Previous studies have shown that the KEGG pathway of DEGs in roots between plastic-film mulching and clean tillage is linked to stilbenoid, diarylheptanoid, and gingerol biosynthesis; however, no stilbene compounds were found in this treatment. In addition, our previous study showed that, under mulching treatment (rice straw + felt + plastic film), grapevine root tissue DEGs were significantly enriched in stilbenoid, diarylheptanoid, and gingerol biosynthesis [5]. Previous studies have shown that stilbenes are involved in grapevine defense responses against fungal pathogens and abiotic stress [43,44,45]. Our previous study found that the plant–pathogen pathway was enriched under mulching [5], which may explain why stilbene-related pathways were enriched after treatment.

Two modules significantly related to stilbene compounds (3,4′-dihydroxy-5-methoxystilbene, piceid, and 1-methyl-resveratrol) were determined by integrated transcriptomic and metabolomic analyses using WGCNA. Two key transcription factors were identified in both the modules. Several transcription factors that are involved in regulating stilbene biosynthesis have been reported [46]. MYBs are among the largest families of plant transcription factors that regulate stilbenes [47]. The Chinese wild grape *VdMYB1* transcription factor might activate the gene expression of stilbenoid biosynthesis enzymes [48]. WRKY and ERF have also been found to regulate stilbene synthesis by interacting with MYB [49,50]. In the present study, HB-HD-ZIP TCP (VIT_13s0156g00260) and TCP (VIT_02s0025g04590) had positive and negative relationships, respectively, with the number of stilbene compounds (3,4′-dihydroxy-5-methoxystilbene, piceid, and 1-methyl-resveratrol). The transcription factors related to stilbene compounds that we identified were different from those identified in previous studies, and the target metabolism differed between the present and previous studies. While the previous study focused on resveratrol synthesis, our study focused on 3,4′-dihydroxy-5-methoxystilbene, piceid, and 1-methyl-resveratrol. Two key seed genes (VIT_09s0054g00610 and VIT_13s0156g00260) were identified in the turquoise and blue modules; however, their function and structure require further investigation. In addition, the results were obtained during the grape maturity period, and all berry development periods should be considered in further research, which would provide more sufficient and comprehensive data for the study of related mechanisms.

Stilbenes, a major family of polyphenols, exhibit a wide range of pharmacological properties and various health benefits [51,52]. In our study, 10 stilbene compounds were detected, which showed a significantly increasing trend after mulch treatment (Figure 3). Resveratrol, ampelopsin F, piceid, ε-viniferintrans-resveratroll, epsilon-viniferin, and pterostilbene possess anticancer, antioxidant, anti-inflammatory, cardiovascular-protective, and anti-aging properties [53,54,55,56,57,58]. Overall, the mulching treatment used in this study may have beneficial effects on grapes.

## Data Availability

The original contributions presented in this study are included in this article/Supplementary Materials; further inquiries can be directed to the corresponding author.

## Supplementary Materials

The following supporting information can be downloaded at https://www.mdpi.com/article/10.3390/foods13193208/s1, Table S1. Fruit quality under the control and mulch treatment; Table S2. Detected Class I compounds; Table S3. Compounds detected from classes other of Class I; Different lowercase letters indicate significant differences among groups (*p* < 0.05) in Tables S1 to S3. Table S4. KEGG enrichment of differential metabolites between the control and mulching; Table S5. Quality control results of transcriptional sequencing; Table S6. KEGG pathway enrichment of DEGs.
